# Adherence to anticoagulation: an ongoing challenge

**DOI:** 10.1007/s12471-019-01347-3

**Published:** 2019-11-21

**Authors:** J. Seelig, M. E. W. Hemels

**Affiliations:** 1 Department of Cardiology, Rijnstate, Arnhem, The Netherlands; 2 Department of Cardiology, Radboud University Medical Centre, Nijmegen, The Netherlands

The introduction of the non-vitamin-K oral anticoagulants (NOACs) has caused a paradigm shift in the treatment of ‘non-valvular’ atrial fibrillation (nvAF). The benefits of a steady daily dosage, its rapid and relatively short-acting character, a non-inferior efficacy profile, and a 50% reduction in intracranial haemorrhages compared to vitamin‑K antagonists (VKAs) are probably the main reasons why NOAC prescription has surpassed VKA prescription in newly diagnosed nvAF patients in the Netherlands [1]. However, when the NOACs were introduced in the Netherlands, concerns were raised about the safety of these drugs in daily clinical practice [2]. One of these concerns was that, with a lack of regular monitoring, NOAC users would be more non-adherent than VKA users [3]. Since then, NOACs have also been shown to be at least non-inferior to VKA therapy in nvAF in various large, real-world cohorts at a European and global level [4]. It should be noted, however, that the well-organised system of anticoagulation clinics in the Netherlands is unique. Comparative data from other countries on adherence, persistence and its relation to thromboembolism or bleeding are therefore difficult to translate into Dutch practice. Moreover, targeting non-adherence in NOAC users, as is usual with VKA users, could further improve outcomes and reduce costs [5, 6].

In this issue, Bennaghmouch et al. [7] report on non-adherence to anticoagulation in AF, using the validated and widely used Morisky medication adherence scale (MMAS-8) for therapy adherence. Also, a questionnaire on practical issues with medication intake, specifically designed for this study, was used. In 2016, 1200 patients using VKAs and 1200 patients using NOACs, from the Star Medical Diagnostic Centre Rotterdam (Star-MDC) and from the St. Antonius Hospital Nieuwegein respectively, were asked to fill out these questionnaires. The primary endpoint was defined as ‘any sort of lack of adherence’, i.e. a MMAS‑8 score of <8 out of a maximum of 8 points. Only 32% of the questionnaires were completed and returned. The authors report that the VKA group had a significantly higher proportion of the primary endpoint than the NOAC group (24.4% vs 18.1%, *p* = 0.03). In the MMAS‑8, patients using VKAs more frequently reported ‘sometimes forgetting to take medication’ (15.4% vs 9.8%, *p* = 0.02) and ‘a hassle to stick to the treatment plan’ (8% vs 4.5%, *p* = 0.05). The newly designed questionnaire on practical issues showed that patients using VKAs more often reported problems with the size and shape of the drugs, opening the blister, changes in the packaging and name of the drug, tablets accidentally crumbling, tablets being too small and, as expected, following a dosage schedule. The authors concluded that it remains unclear to what extent these practical issues lead to non-adherence in daily clinical practice.

Bennaghmouch and co-workers are to be complimented on performing this research. In contrast to the hypothesis of ‘less monitoring leads to more non-adherence’, this study showed that any lack of adherence according to the MMAS‑8 was more often present in the VKA group than in the NOAC group. Unfortunately, it is often difficult to determine therapy non-adherence accurately. Generally, two methods are used to determine non-adherence in observational research, namely (1) using questionnaires, or (2) using pharmacy dispensing data [8]. Both methods have their limitations, and one method is not generally better than the other, but they are complementary. There are, of course, other more accurate ways to determine non-adherence, such as pill count or measuring drug serum levels, but these methods are difficult to implement on a large scale. Regarding adherence questionnaires, the MMAS‑8 is probably the most widely accepted, but there is no adherence questionnaire which is considered ‘the best’, and its use is limited [8]. A meta-analysis of 28 studies using the MMAS‑8 showed that with a cut-off value of < 6 points, specificity for non-adherence was moderate [0.73, 95% confidence interval (CI) 0.68–0.78] and sensitivity was low (0.43, 95% CI 0.33–0.53) [9]. The test properties with a cut-off value of <8 as used by Bennaghmouch et al. have been studied less frequently. In a Korean study in patients with diabetes, a cut-off value of <8 versus <6 resulted in an increased sensitivity (73.8% vs 48.7%) and decreased specificity (38.1% vs 69.1%) [10]. However, this resulted in a poorer positive predictive value (69.7% vs 63.9%) for non-adherence and a consistently poor negative predictive value (47.6% vs 50%).

Given the limitations of adherence questionnaires and the low response rate, it is difficult to draw strong conclusions from this study. Bennaghmouch et al. [7] provided an important first step, but more research, preferably with a combination of adherence questionnaires or questionnaires complemented with pharmacy dispensing data, is needed [8]. Moreover, there has not yet been sufficient research on the impact of anticoagulation non-adherence on the incidence of stroke and other adverse events in patients with nvAF. The growing incidence of bleeding due to an aging population using anticoagulants is another factor that will challenge adherence in the near future. The ongoing DUTCH-AF registry (ZonMw project numbers 848050006 and 848050007) is a large prospective study in the Netherlands that uses a multi-measure approach for non-adherence, and it is powered to relate these results to adverse events. We hope that this study will give further insights into (non-)adherence to anticoagulation and will provide us with the tools needed to improve treatment in daily clinical practice accordingly.
